# How *Should* Youth Handgrip Strength be Normalized? New Insights Using 3-D Allometry with “Generalizable” Norm-Referenced Values, Data from NHANES

**DOI:** 10.1007/s40279-025-02235-0

**Published:** 2025-05-30

**Authors:** Alan M. Nevill, Justin J. Lang, Mark Niemz, Grant R. Tomkinson

**Affiliations:** 1 Faculty of Education, Health and Wellbeing, University of Wolverhampton, Walsall Campus, Walsall, UK; 2 Centre for Surveillance and Applied Research, Public Health Agency of Canada, Ottawa, ON Canada; 3 School of Epidemiology and Public Health, Faculty of Medicine, University of Ottawa, Ottawa, ON Canada; 4 Alliance for Research in Exercise, Nutrition and Activity (ARENA), Allied Health and Human Performance, University of South Australia, GPO Box 2471, Adelaide, SA 5001 Australia

## Abstract

**Background:**

Handgrip strength (HGS) is an important marker of health. Using allometric scaling, we previously identified that adult HGS should be normalized by a cross-sectional or surface area measure of body size, although it is unclear whether scaling youth HGS by the same body size dimension is appropriate. We therefore aimed to (1) identify the optimal body size dimension(s) to normalize youth HGS for differences in body size and (2) generate norm-referenced values for HGS using the identified body size dimension(s).

**Methods:**

Data were from the National Health and Nutrition Examination Survey (NHANES), a representative sample of the US non-institutionalized civilian population. Exclusions resulted in a final sample of 4816 youth (51.2% male) aged 6–19 years. Handgrip strength was measured using electronic hand dynamometry. Body size dimensions included body mass, height, and waist circumference. Allometry was used to identify the most appropriate dimension(s) associated with HGS. Population-weighted, sex-stratified generalized additive models for location, scale, and shape were used to develop norms by sex and age. Norms were tabulated as percentile values (3rd to 97th) and visualized as smoothed percentile curves.

**Results:**

Predicting HGS using all three body size dimensions (three-dimensional) resulted in collinearity predominantly owing to the presence of waist circumference, prohibiting the use of all three body size dimensions as normalizers. However, collinearity was not an issue when two of the three dimensions (body mass and height) were adopted. Allometry identified a “generalizable” normalizing ratio as HGS_n_ = $HGS/({HT}^{2}*{M}^{0.333})$. If only a single body size dimension were available, then HGS should be normalized using height^2^ (i.e., $HGS/{HT}^{2}$) because height was identified as the strongest single body size dimension associated with HGS. Sex- and age-specific norms for $HGS/({HT}^{2}*{M}^{0.333})$ declined from age 6–8 years and progressively increased thereafter.

**Conclusions:**

Allometrically scaling HGS by $({HT}^{2}*{M}^{0.333})$ helps normalize strength for body size in population-based youth research.

**Supplementary Information:**

The online version contains supplementary material available at 10.1007/s40279-025-02235-0.

## Key Points

**Table:** 

Handgrip strength (HGS) is an important marker of health. While body size is known to influence HGS, the optimal body size dimension(s) to normalize youth HGS for differences in body size is unclear.
Using the dimensions of height (*HT*) and body mass (*M*), allometry identified a “generalizable” normalizing ratio for youth as HGS_n_ = $HGS/({HT}^{2}*{M}^{0.333})$. If only a single body size dimension were available, then HGS should be normalized by height-squared (i.e., $HGS/{HT}^{2}$).
Allometrically scaling HGS by $({HT}^{2}*{M}^{0.333})$ helps normalize strength for body size in population-based youth research.

## Introduction

Muscular strength is often measured using isometric hand dynamometers to assess handgrip strength (HGS), which is considered an important marker of health among youth [1, 2]. Assessing HGS in schools is easy, feasible, and recommended for its predictive utility by the National Academy of Medicine (formerly the Institute of Medicine) [3]. Assessments of HGS are highly reliable [4] and valid [5] when compared to whole-body and large muscle group strength assessments. Handgrip strength was recently recommended by international experts as a key test for youth fitness monitoring and surveillance [6]. In addition, the dynamometers used to assess HGS are becoming more affordable, with evidence demonstrating comparable results between the lower cost and standard (e.g., TKK) devices [7]. These advances will greatly improve the accessibility of these measures in lower socioeconomic communities.

It is well documented that heavier individuals often have a strength advantage in assessments that do not involve supporting body mass, such as HGS [8, 9]. For this reason, there is a strong positive association between absolute HGS performance and adiposity [1]. Indeed, this is counterintuitive and does not align with HGS as a health-related fitness measure. To help partition the effects of body mass from HGS results, some researchers have used ratio scaling to calculate a measure of HGS relative (i.e., normalized) to body size. Early research by Vanderburgh et al. [10] demonstrated that ratio scaling (i.e., where HGS was divided by body mass) was no better at partitioning out the effect of body mass than absolute HGS. For this reason, allometric scaling has emerged as a preferred option for normalizing HGS. For example, allometric models have been adopted by Nevill et al. [11] and Bustamante Valdivia et al. [12] when adjusting HGS for differences in body mass and height of children from Greece and Peru, respectively. Allometric scaling has also been used to normalize other functional measures like muscular power (e.g., Wingate cycling tests, isokinetic power assessments), rate of force development (e.g., maximum slope of the force–time curve), and movements requiring high force to overcome body weight (e.g., pull-ups, push-ups) [8].

More recently, Kocher et al. [13] applied allometric scaling to a large nationally representative sample of American youth aged 6–18 years and successfully partitioned the effects of body size on HGS, as demonstrated by non-statistically significant correlation coefficients with body mass of near 0. The allometric exponents identified by Kocher et al. [13] were sex and age specific, which by their own admission makes it impossible to compare the normalized HGS among youth who differ by sex or age because of differences in the fitted mass and height exponents. Another limitation associated with Kocher’s sex- and age-specific normalizing equations [13] is that if we were to replicate their methods on American youth a decade later, or indeed another population such as Canadian youth, their fitted exponents would differ. With different populations, any subsequent examination of Kocher’s [13] exponents, reported to have passed both the regression diagnostics and Vanderburgh’s “litmus test”, would now almost certainly violate such diagnostics/tests using new/independent samples/populations. We have previously demonstrated that a single “theoretically sound” allometric scaling exponent could be effective at partitioning out body size when measuring HGS among American adults [14]. Exploring this approach among youth may help improve the research originally conducted by Kocher et al. [13], while greatly advancing the health-related utility of HGS.

Thus, using a nationally representative sample of American youth aged 6–19 years, we aimed to: (a) identify, using allometric scaling, the optimal body size dimension(s) to normalize youth HGS for differences in body size and (b) generate norm-referenced values for body size-normalized HGS estimated using the Generalized Additive Model for Location, Scale, and Shape (GAMLSS) [15]. Based on the fitted body mass and height exponents reported by Nevill et al. [11] and Bustamante Valdivia et al. [12] when predicting HGS of Greek and Peruvian children, respectively, we hypothesized that the most appropriate body size dimension associated with HGS was likely to be either a cross-sectional area [12] or a volumetric measure [11] of body size.

## Methods

### Participants

Data from the 2011–2012 and 2013–2014 cycles of the National Health and Nutrition Examination Survey (NHANES) dataset were used because they included measures of HGS. The NHANES uses a complex multi-stage probability design to assess a representative sample of the US non-institutionalized civilian population [16]. The NHANES included an in-person home interview and a visit to a nearby mobile examination center for medical, physiological, and laboratory measures. Signed parental consent (participants aged 0–6 years), signed parental consent and child assent (participants aged 7–17 years), and signed consent (participants aged 18 + years) were provided as part of the original NHANES data collection. The NHANES protocols (Protocol #2011–17) were approved by the National Center for Health Statistics Research Ethics Review Board in compliance with the revised Declaration of Helsinki. We did not seek additional approval because the data used in this study were free from personal identifiers.

We only used data on youth aged 6–19 years in this study. Of the initial 19,931 participants, 15,115 were excluded because they: (a) were aged 20 years or older (*n* = 14,527); (b) did not complete HGS testing on both hands (*n* = 457); and (c) had missing data (e.g., HGS, body mass, height, waist circumference) [*n* = 287]. These exclusions resulted in a final sample of 4816 youth (51.2% male) aged 6–19 years.

### Measures

The standardized HGS and anthropometry protocols are described in detail elsewhere [17–20]. Handgrip strength was measured using a Takei electronic hand dynamometer (Model T.K.K.5401; Takei Scientific Instruments, Niigata, Japan). A trained examiner explained and demonstrated the HGS protocol. Following adjustment of the dynamometer for hand size, participants performed a sub-maximal effort practice trial to check that the dynamometer was fitted properly, and the procedure was understood. Participants randomly started the HGS test with their right or left hand, and while standing upright with their feet hip width apart, their arm extended and hanging down away from their body, they squeezed the dynamometer with maximal effort while exhaling. Three trials were performed for each hand, alternating hands between trials, with 60 s of rest between measures on the same hand. The typical error across the three trials was 8.0% (95% confidence interval [CI] 7.9, 8.1) or 1.70 kg (95% CI 1.68, 1.72). Handgrip strength was taken as the maximum score irrespective of the hand because it best aligns with strength capacity [21].

Trained examiners measured the body mass of participants with a digital weighing scale (Mettler-Toledo, Columbus, OH, USA). Height was measured with a fixed stadiometer and adjustable headboard. Waist circumference was measured at end-tidal expiration using a steel measuring tape positioned in a horizontal plane at the top of the iliac crests. Routine calibration checks of all equipment were performed as part of the quality-control procedures to ensure that the equipment produced accurate measurements. Participants self-reported their sex and age.

### Statistical Analyses

All statistical analyses were conducted in IBM SPSS Statistics (v26, IBM; Chicago, IL, USA), except for the norms that were generated in R (v4.4.0) using the GAMLSS package [15]. To obtain nationally representative estimates, analyses were conducted using NHANES sample weights (survey, strata, and cluster weights), which account for the complex survey design (including oversampling), survey non-response, and post-stratification. We adopted the following three-dimensional multiplicative model with allometric body size components (Eq. 1), like that used to model the HGS of American adults [14], to identify the most appropriate body size dimension(s) associated with HGS among American youth.

1 $$HGS=a\cdot {M}^{{k}_{1}}\cdot {HT}^{{k}_{2}}\cdot {WC}^{{k}_{3}}\cdot \varepsilon ,$$

where ‘$a$’ is the scaling constant and ${k}_{1}$, ${k}_{2}$, and ${k}_{3}$ are scaling exponents for the body mass ($M$), height ($HT$), and waist circumference ($WC$) respectively, and $\varepsilon$ is the multiplicative error ratio [22]. The multiplicative error ratio ‘$\varepsilon$’ assumes that the error increases in proportion to body size, a characteristic in data known as heteroscedasticity, which can be adjusted statistically by taking logarithms as described below. Sex and age were incorporated into the model by allowing ‘$a$’ to vary for either sex by age (discrete ages 6, 7, 8 … 19 years) to accommodate the likelihood that HGS may rise more steeply in boys compared with girls. This model can be linearized by log-transformation, and multiple regression/analysis of covariance (ANCOVA) can be used to estimate the body mass, height, and waist circumference exponents for HGS having controlled for both sex and age (Eq. 2). Log-transformed HGS effectively becomes the dependent variable, with sex and age as the fixed factors and log($M$), log($HT$), and log(WC) the covariates.

2 $$log(HGS)=log(a) + {k}_{1}\cdot log(M)+{k}_{2}\cdot log(HT)+{k}_{3}\cdot log(WC)+log(\varepsilon )$$

Partial eta-squared (*η*^2^) was used as the measure of effect size. An alpha level of 0.05 was used for the analyses.

We developed norms using GAMLSS by fitting 50 response distributions and three nonparametric smoothing functions (i.e., cubic splines, polynomial splines, and fractional polynomials). All models were stratified by sex. Detrended Q-Q plots (worm plots) were used for visual inspection. The best fitting models were selected using Akaike Information Criterion values. The 3rd, 5th, 10th, 20th, 30th, 40th, 50th, 60th, 70th, 80th, 90th, 95th, and 97th percentiles were calculated by sex and age. Norms were tabulated as percentiles values and visualized as smoothed percentile curves.

## Results

**Fig. 1:**
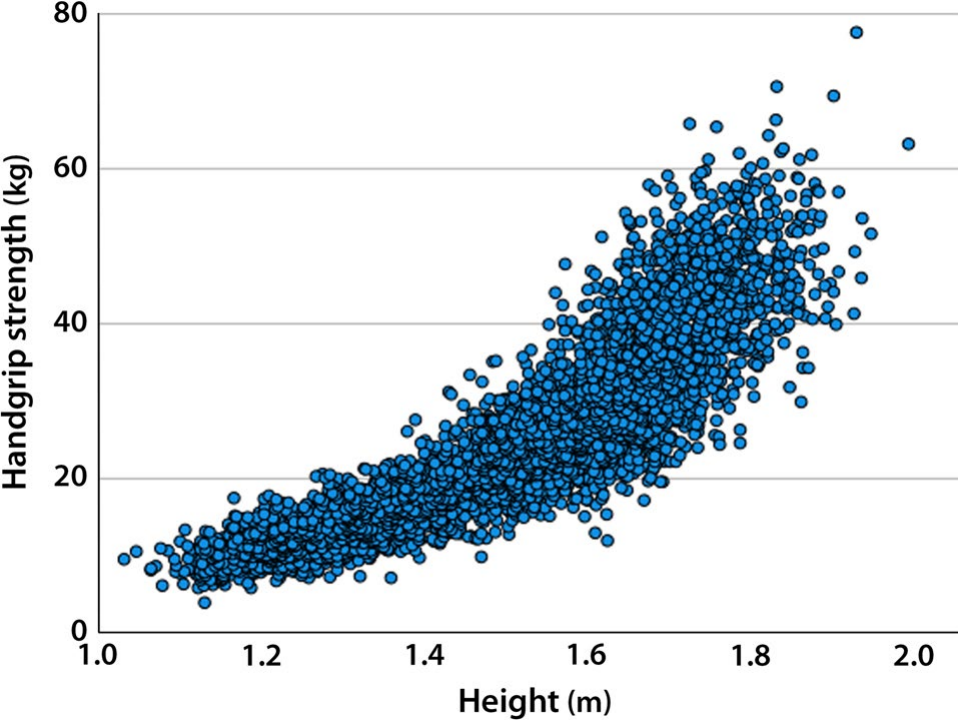
Association between handgrip strength (kilogram [kg]; maximum score attained irrespective of the hand) and height (m)

Figure 1 shows the very strong and positive relationship between HGS and body size (i.e., height) among American youth (*r* [95% CI] = 0.88 [0.87, 0.89]). This figure shows that the errors increase with height (i.e., heteroscedasticity), which we statistically adjusted by taking logarithms as described in the Methods section. Note, correlations between HGS and both body mass (*r* [95% CI] = 0.78 [0.77, 0.79]) and waist circumference (*r* [95% CI] = 0.59 [0.57, 0.61]) were significantly weaker than that for height.

**Fig. 2:**
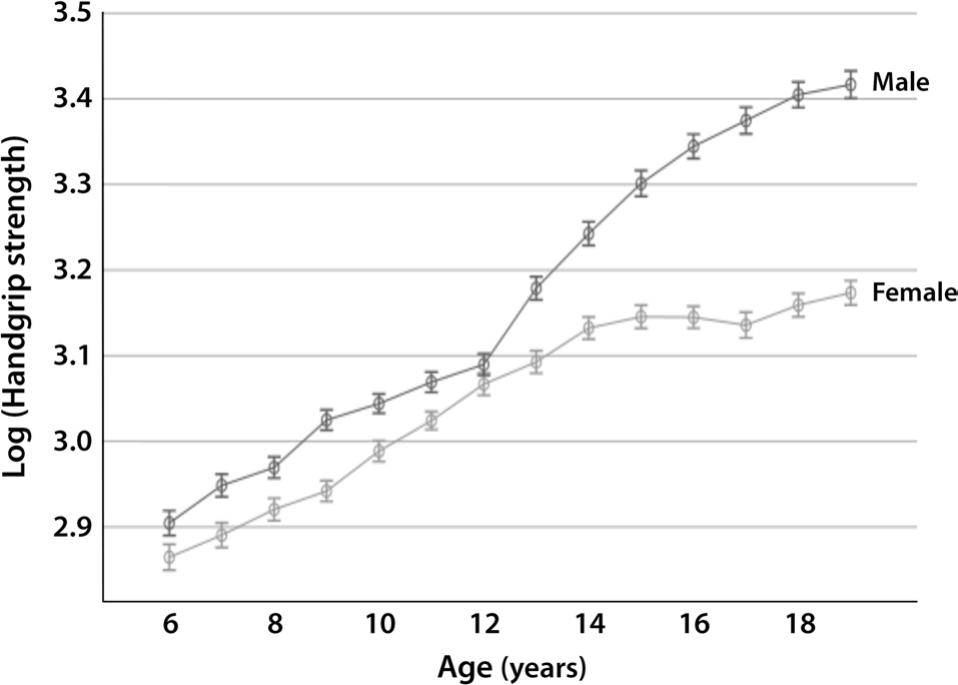
Means (± standard errors) of log-transformed handgrip strength adjusted for log($M$), log($HT$), and log($WC$) by sex and age. *HT* height, *M* body mass, *WC* waist circumference

The ANCOVA of log-transformed HGS incorporating the three body size covariates described in Eq. 2 identified significant main effects for sex and age, and a significant age-by-sex interaction (*p* < 0.001). These effects can be seen in Fig. 2.

The ANCOVA also revealed that all three log-transformed body size covariates were significant (Table 1). Note that the fitted body mass and height exponents are positive, but the waist circumference exponent is negative, confirming that greater body mass and height benefit HGS but excess waist circumference is harmful to HGS. The explained variance of the ANCOVA identified an *R*^2^ = 0.897 (adjusted *R*^2^ = 0.896), assuming common exponents for all three body size covariates by sex and age. We found a trivial increase in explained variance when the three body size exponents were allowed to vary by age group (*n* = 14) and sex (*R*^2^ = 0.899, adjusted *R*^2^ = 0.897), requiring an additional 3*(13 + 1) = 42 degrees of freedom.

**Table 1:** Fitted parameters of the analysis of covariance predicting log-transformed hand grip strength using all three body size covariates

Parameter	*B*	*SE*	*t*	*p*	95% Confidence interval		*η*^2^
					Lower bound	Upper bound	
Log($M$)	0.982	0.033	29.8	< 0.001	0.917	1.047	0.16
Log($HT$)	0.609	0.073	8.4	< 0.001	0.466	0.752	0.01
Log($WC$)	− 1.096	0.046	− 24.0	< 0.001	− 1.186	− 1.007	0.11
Female	− 0.243	0.020	− 12.4	< 0.001	− 0.282	− 0.205	0.03

*R*^2^ = 0.897 (Adjusted *R*^2^ = 0.896)

*B* regression coefficient, *HT* height, *M* body mass, *η*^2^ partial eta-squared, *p* probability values, *SE* standard error, *t* t value, *WC* waist circumference

Unfortunately, the ANCOVA reported in Table 1 identified strong evidence of collinearity owing to the presence of log($WC$). This prohibits the use of all three body size covariates when predicting and hence normalizing youth HGS. As such, we re-ran the ANCOVA predicting log(*HGS*) using only log($M$) and log($HT$) as covariates. The fitted parameters are given in Table 2. The explained variance of the ANCOVA reported in Table 2 identified an *R*^2^ = 0.884 (adjusted *R*^2^ = 0.884).

**Table 2:** Fitted parameters of the analysis of covariance predicting log-transformed handgrip strength adopting the log-transformed body mass and height size as covariates

Parameter	*B*	*SE*	*t*	*p*	95% Confidence interval		*η*^2^
					Lower bound	Upper bound	
Log($M$)	0.236	0.012	20.2	< 0.001	0.213	0.259	0.08
Log($HT$)	1.586	0.064	24.8	< 0.001	1.461	1.712	0.11
Female	− 0.271	0.021	− 13.1	< 0.001	− 0.311	− 0.230	0.03

*R*^2^ = 0.884 (adjusted *R*^2^ = 0.884)

*B* regression coefficient, *HT* height, *M* body mass, *η*^2^ partial eta-squared, *p* probability values, *SE* standard error, *t* t value

Finally, because height was found to be the strongest single predictor of HGS, we re-ran the ANCOVA using a simplified/reduced allometric model (Eq. 2), excluding log($M$) and log($WC$). This follow-up analysis revealed the height covariate as highly significant (Table 3), with the fitted height ($HT$) exponent greater than 2. The curvature can be clearly seen in Fig. 1. The explained variance of the ANCOVA reported in Table 3 identified an *R*^2^ = 0.875 (adjusted *R*^2^ = 0.874).

**Table 3:** Fitted parameters of the analysis of covariance predicting log-transformed handgrip strength adopting only the log-transformed height body size as a covariate

Parameter	*B*	*SE*	*t*	*p*	95% Confidence interval		*η*^2^
					Lower bound	Upper bound	
Log($HT$)	2.351	0.054	43.7	< 0.001	2.245	2.456	0.29
Female	− 0.250	0.022	− 1.6	< 0.001	−.292	− 0.207	0.03

*R*^2^ = 0.875 (adjusted *R*^2^ = 0.874)

*B* regression coefficient, *HT* height, *η*^2^ partial eta-squared, *p* probability values, *SE* standard error, *t* t value

**Fig. 3:**
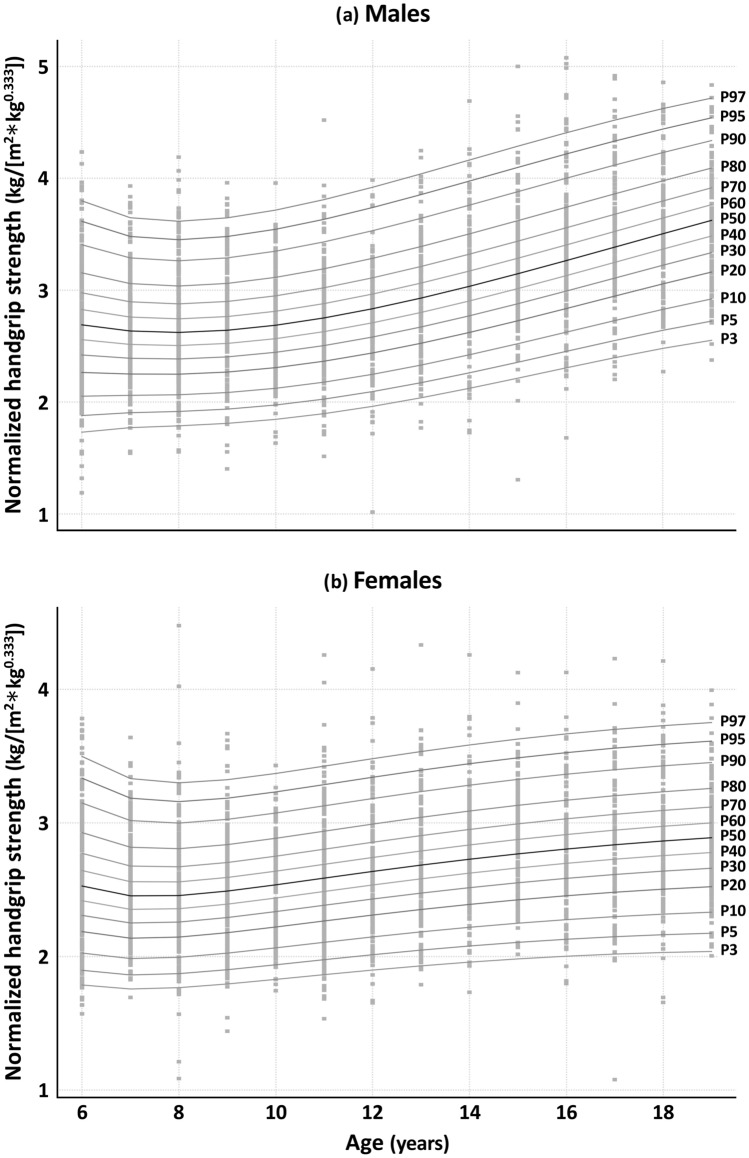
Percentile curves for normalized handgrip strength (handgrip strength in kilograms [kg] divided by [height in meters (m) squared multiplied by body mass in kg raised to the power of one-third]) by age for (**a**) males and (**b**) females. *P* percentile (e.g., P5 = 5th percentile)

The sex- and age-specific norms for the two-dimensional normalized HGS_n_ given by $HGS/({HT}^{2}*{M}^{0.333})$ are tabulated as percentiles values (Table 4) and visualized as smoothed percentile curves (Fig. 3). Note that the adopted $HT$ and $M$ exponents of 2 and 0.333, respectively are justified in the Discussion but are chosen predominantly to allow our recommended HGS_n_ ratio to be generalized to, and compared with, other populations. Similarly, the corresponding simplified norms for height alone given by $HGS/{(HT}^{2}),$ are given in Table S1 and Fig. S1 of the Electronic Supplementary Material. The norms given in Table 4 and Fig. 3 using $HGS/({HT}^{2}*{M}^{0.333})$ can be used to estimate an individual’s normalized HGS using a nationally representative sample of Americans aged 6–19 years for comparative purposes.

**Table 4:** Norm-referenced values (percentiles) for normalized handgrip strength (handgrip strength in kilograms divided by [height in meters squared multiplied by body mass in kilograms raised to the power of one-third]) by sex and age for a nationally representative sample of Americans aged 6–19 years

Age (y)	P_3_	P_5_	P_10_	P_20_	P_30_	P_40_	P_50_	P_60_	P_70_	P_80_	P_90_	P_95_	P_97_
*Male*													
6	1.721	1.875	2.055	2.276	2.439	2.584	2.725	2.871	3.031	3.223	3.494	3.721	3.919
7	1.782	1.918	2.076	2.270	2.414	2.542	2.666	2.796	2.938	3.108	3.349	3.550	3.726
8	1.807	1.936	2.085	2.270	2.407	2.528	2.647	2.770	2.906	3.069	3.300	3.492	3.661
9	1.830	1.956	2.103	2.285	2.420	2.540	2.658	2.780	2.915	3.077	3.305	3.497	3.664
10	1.862	1.989	2.136	2.319	2.455	2.575	2.694	2.817	2.954	3.117	3.349	3.543	3.712
11	1.907	2.036	2.185	2.371	2.508	2.631	2.752	2.878	3.017	3.184	3.420	3.618	3.791
12	1.967	2.098	2.250	2.439	2.580	2.705	2.829	2.958	3.100	3.271	3.512	3.715	3.892
13	2.040	2.174	2.330	2.524	2.668	2.796	2.922	3.054	3.200	3.375	3.622	3.829	4.010
14	2.125	2.262	2.423	2.622	2.770	2.901	3.030	3.165	3.314	3.492	3.745	3.957	4.142
15	2.220	2.362	2.528	2.732	2.884	3.019	3.151	3.288	3.440	3.622	3.879	4.094	4.282
16	2.324	2.471	2.642	2.853	3.009	3.147	3.282	3.422	3.576	3.760	4.021	4.238	4.429
17	2.434	2.587	2.764	2.982	3.143	3.284	3.422	3.564	3.720	3.906	4.168	4.387	4.578
18	2.548	2.707	2.892	3.118	3.284	3.429	3.569	3.712	3.870	4.056	4.319	4.538	4.729
19	2.663	2.829	3.023	3.258	3.430	3.579	3.722	3.867	4.024	4.211	4.472	4.690	4.879
*Females*													
6	1.794	1.908	2.041	2.209	2.336	2.452	2.569	2.693	2.831	2.999	3.237	3.438	3.614
7	1.765	1.874	2.001	2.160	2.279	2.387	2.494	2.608	2.733	2.884	3.099	3.280	3.439
8	1.775	1.884	2.010	2.167	2.285	2.390	2.494	2.603	2.723	2.868	3.074	3.246	3.397
9	1.803	1.913	2.040	2.199	2.316	2.421	2.524	2.632	2.751	2.893	3.095	3.264	3.412
10	1.837	1.948	2.079	2.239	2.358	2.464	2.567	2.675	2.794	2.935	3.136	3.304	3.450
11	1.871	1.986	2.119	2.282	2.403	2.510	2.614	2.722	2.841	2.983	3.183	3.351	3.497
12	1.904	2.021	2.157	2.324	2.448	2.556	2.661	2.770	2.889	3.031	3.232	3.399	3.546
13	1.934	2.054	2.193	2.364	2.489	2.599	2.705	2.815	2.934	3.077	3.278	3.445	3.592
14	1.960	2.083	2.225	2.399	2.527	2.639	2.746	2.856	2.976	3.119	3.320	3.488	3.634
15	1.982	2.108	2.253	2.431	2.561	2.674	2.782	2.893	3.014	3.157	3.358	3.525	3.671
16	2.000	2.128	2.277	2.459	2.591	2.706	2.815	2.926	3.047	3.191	3.391	3.558	3.703
17	2.013	2.145	2.297	2.482	2.617	2.734	2.844	2.956	3.077	3.220	3.419	3.585	3.729
18	2.021	2.156	2.312	2.502	2.639	2.757	2.869	2.981	3.102	3.244	3.443	3.607	3.750
19	2.025	2.164	2.323	2.517	2.657	2.777	2.889	3.002	3.123	3.265	3.462	3.625	3.766

Norm-referenced values are in kg/(m^2^ $*$ kg^0.333^); population-weighted smoothed percentiles were calculated using the Generalized Additive Model for Location, Scale, and Shape method; the ages shown represent age (e.g., 6 = 6.00–6.99 …) based on age at last birthday

*P* percentile (e.g., P5 = 5th percentile), *y* years

## Discussion

With a positive height and a negative waist circumference exponent (Table 1), the resulting “inverted” waist-to-height ratio appears to be a valuable predictor of youth HGS—the greater the “excess” waist-to-height ratio, the lower the HGS. However, attempts to fit a three-dimensional multiplicative allometric model for log($HGS$) [Eq. 2] using all three body size terms were thwarted owing to collinearity caused by the presence of waist circumference (i.e., by including the combination of the body mass and height terms, the contribution of waist circumference appeared redundant). This prohibits the use of all three body size covariates to predict, and hence normalize, HGS for differences in body size.

In addition to the problem of collinearity discussed above, we recognize that waist circumference might not always be available to normalize HGS in pediatric studies. For this reason, we identified how HGS should be normalized for body mass and height, with the fitted exponents reported in Table 2. The resulting normalized HGS_n_ becomes,

3 $${HGS}_{n}=\frac{HGS}{{M}^{0.236}\cdot {HT}^{1.586}}.$$

Previous research by Kocher et al. [13] reported norms for HGS using the same 2011–12 and 2013–2014 NHANES data set, adopting a similar allometric model to that reported in Eq. 3. The major difference between our approach and that adopted by Kocher et al. [13] is that they fitted *different* mass and height exponents for *all* age groups and *both* sexes. The authors recognized this as a major limitation of their work when reporting the normalized percentile values for HGS by sex and age in their Tables 6 and 7, stating that it was “inappropriate to make comparisons between age groups and sexes as the allometric exponents differ” by sex and age [13]. However, allowing the body mass and height exponents to vary by sex and age resulted in a trivial increase in adjusted *R*^2^ values. This supports the use of our “common exponent” model that fitted the same body mass and height exponents across all ages and both sexes, as estimated in Table 2. The effect sizes reported in Table 2 confirm that height (taller youth have a mechanical advantage of greater leverage) is a more important (effective) predictor of youth HGS than body mass. This was also confirmed in adults by Nevill and Holder [23].

The final “simplified” ANCOVA model adopting just a single body size dimension, log($HT$) as the covariate to predict log($HGS$), revealed that to obtain a normalized HGS_n_ independent of height, we need to calculate the normalized ratio,

4 $${HGS}_{n}=HGS/({HT}^{2.351})$$

The fitted exponents in the models reported in Tables 2 and 3 are not entirely compatible from dimensional considerations, as anticipated by Åstrand and Rodahl [23]. In their chapter on body dimensions and muscular exercise, Åstrand and Rodahl [23] report that force should scale to the physiological dimension of ${L}^{2}$, where $L$ is a linear dimension of body size. Using $L$ as the common linear body size dimension (e.g., body mass, $M={L}^{3}$), the HGS denominator of Eq. 3 becomes $\left({M}^{0.236}\cdot {HT}^{1.586}\right)={\left({L}^{3}\right)}^{0.236}\cdot { L}^{1.586}={L}^{0.708}\cdot {L}^{1.586}$ $={L}^{2.294}$, or approximately ${L}^{2.3}$. This is slightly higher than that anticipated by Åstrand and Rodahl [23], suggesting that youth HGS is associated with, or proportional to a cross-sectional area (L^2^), plus a further advantage of approximately ${L}^{0.3}$, possible owing to the benefit of having a greater leverage obtained from being taller. The same conclusion can be inferred from Eq. 4, when we modeled log($HGS$) using just the single body size dimension log($HT$). Table 3 confirms that the height exponent was 2.351, also slightly higher than 2.

Adopting similar arguments to those discussed by Nevill et al. [14] when normalizing adult HGS, the fitted exponents adopted and reported here for youth in Eqs. 3 and 4 are all “sample specific”. That is, they are likely to work well for American youth, but these exponents will never be duplicated/reproduced exactly in other youth populations, either within or outside the USA. As such, Nevill et al. [14] argued that, when normalizing adult HGS, a *simple* methodology that is likely to be physiologically sound but also “generalizable” to all populations is needed. The same strategy applies here when recommending the most appropriate methodology to normalize youth HGS.

Our two-dimensional allometric model (Eq. 3) used to normalize youth HGS incorporating two of the body size dimensions of height and mass suggests that we should adopt a normalizing term of approximately ${HT}^{2}$ (that will reflect an advantage of being taller) multiplied by a small additional body mass component, which contributes a small additional advantage of having greater body mass, approximately ${M}^{0.333}$(or L). As such, provided both body size dimensions are available, we recommend a generalizable HGS be normalized by dividing HGS by $({HT}^{2}*{M}^{0.333})$ or ${HGS}_{n}$= $HGS/({HT}^{2}*{M}^{0.333}).$ However, if only a single body size dimension were available, given we confirmed that height was the single best body size dimension associated with HGS, then we recommend that HGS be normalized by dividing HGS by height (${HT}^{2}$). This “simple” solution would then follow, and be compatible with, a recommendation that was proposed by Nevill et al. [14] for optimally normalizing *adult* HGS.

Our findings have several implications for sports medicine and exercise science professionals. First, studies investigating the health-related predictive utility of youth HGS have typically used absolute HGS rather than normalized HGS [25, 26]. Future studies should examine whether normalized HGS impacts these health-related associations. Second, to compare among youth HGS studies, we recommend reporting both absolute HGS (i.e., in the measured units) and normalized HGS values where possible. Last, without universally accepted health-related cut-points for youth HGS [25], we recommend using a quintile framework to help interpret our HGS percentiles, similar to previous studies [e.g., 14, 27, 28]. For example, youth below the 20th percentile can be considered as having “low” strength; between the 20th and 39th percentiles as having “somewhat low” strength; between the 40th and 59th percentiles as having “moderate” strength; between the 60th and 79th percentiles as having “somewhat high” strength; and at or above the 80th percentile as having “high” strength.

Strengths of our study included the large nationally representative sample across two measurement cycles, and the use of objective HGS and anthropometric measures that were collected by trained examiners using standardized testing protocols. The three-dimensional multiplicative allometric model, which fit the same body mass and height exponents across all ages and both sexes, allowed for a generalizable normalizing ratio for youth HGS. Our use of the NHANES sample weights resulted in population representative estimates. However, this study was limited to cross-sectional survey data, which meant that causality could not be inferred. Further research using a longitudinal study design is required to confirm our findings. Another limitation was that our findings were based on a secondary analysis of NHANES data, and while these data are robust, our analysis was only based on the collected measures.

## Conclusions

Using a large nationally representative sample of American children and youth, we identified a generalizable approach to allometrically scale HGS_n_ by dividing absolute HGS by $({HT}^{2}*{M}^{0.333})$ to help partition the effects of body size. In the absence of mass or if researchers wished to use only one body size dimension, we propose using a simplified scaling approach of HGS divided by (${HT}^{2}$). Future research should explore these scaling approaches in different populations to evaluate their generalizability. Research should also investigate the associations between these allometrically scaled HGS values and health outcomes in youth.

## Supplementary Information

Below is the link to the electronic supplementary material.

Supplementary file1 (DOCX 470 KB)

## Declarations

### Availability of Data and Material

Data are publicly available from the Centers for Disease Control and Prevention, National Center for Health Statistics (https://wwwn.cdc.gov/nchs/nhanes/continuousnhanes/default.aspx?BeginYear=2011, https://wwwn.cdc.gov/nchs/nhanes/continuousnhanes/default.aspx?BeginYear=2013).
