# Supplementary material for: How Should Youth Handgrip Strength be Normalized? New Insights Using 3-D Allometry with “Generalizable” Norm-Referenced Values, Data from NHANES
Source: Sports Med. 2025 May 30;55(9):2303–12. doi: 10.1007/s40279-025-02235-0 (PMC12476433; doi:10.1007/s40279-025-02235-0)

**ELECTRONIC SUPPLEMENTARY MATERIAL**

**Article title:** How *should* youth handgrip strength be normalized? New insights using 3-D allometry with “generalizable” norm-referenced values, data from NHANES; **Journal name:** Sports Medicine; **Author names and affiliations:** Alan M Nevill (University of Wolverhampton), Justin J. Lang (Public Health Agency of Canada, University of Ottawa, and University of South Australia), Mark Niemz (University of Wolverhampton), Grant R. Tomkinson (University of South Australia); **E-mail address of the corresponding author:** [grant.tomkinson@unisa.edu.au](mailto:grant.tomkinson@unisa.edu.au).

**Table S1.** Norm-referenced values (percentiles) for normalized handgrip strength (handgrip strength in kilograms divided by height in meters squared) by sex and age for a nationally representative sample of Americans aged 6–19 years.

| **Age (y)** | **P_3_** | **P_5_** | **P_10_** | **P_20_** | **P_30_** | **P_40_** | **P_50_** | **P_60_** | **P_70_** | **P_80_** | **P_90_** | **P_95_** | **P_97_** |
| --- | --- | --- | --- | --- | --- | --- | --- | --- | --- | --- | --- | --- | --- |
| *Males* |  |  |  |  |  |  |  |  |  |  |  |  |  |
| 6 | 4.9 | 5.3 | 5.7 | 6.3 | 6.7 | 7.1 | 7.5 | 8.0 | 8.5 | 9.1 | 9.9 | 10.5 | 11.1 |
| 7 | 5.3 | 5.7 | 6.1 | 6.7 | 7.1 | 7.5 | 7.9 | 8.3 | 8.7 | 9.3 | 10.0 | 10.7 | 11.2 |
| 8 | 5.7 | 6.1 | 6.5 | 7.1 | 7.5 | 7.9 | 8.3 | 8.7 | 9.1 | 9.6 | 10.4 | 11.0 | 11.5 |
| 9 | 6.0 | 6.5 | 7.0 | 7.6 | 8.0 | 8.4 | 8.8 | 9.2 | 9.6 | 10.1 | 10.9 | 11.5 | 12.1 |
| 10 | 6.4 | 6.9 | 7.4 | 8.1 | 8.5 | 8.9 | 9.3 | 9.7 | 10.1 | 10.7 | 11.5 | 12.2 | 12.9 |
| 11 | 6.8 | 7.3 | 7.9 | 8.6 | 9.1 | 9.5 | 9.9 | 10.3 | 10.8 | 11.4 | 12.3 | 13.1 | 13.8 |
| 12 | 7.2 | 7.8 | 8.4 | 9.2 | 9.7 | 10.1 | 10.5 | 11.0 | 11.5 | 12.1 | 13.1 | 14.0 | 14.8 |
| 13 | 7.7 | 8.3 | 9.0 | 9.8 | 10.3 | 10.8 | 11.2 | 11.7 | 12.2 | 12.9 | 14.0 | 15.0 | 15.9 |
| 14 | 8.2 | 8.9 | 9.6 | 10.4 | 11.0 | 11.5 | 11.9 | 12.4 | 13.0 | 13.7 | 14.9 | 16.0 | 16.9 |
| 15 | 8.8 | 9.5 | 10.2 | 11.0 | 11.7 | 12.2 | 12.7 | 13.2 | 13.8 | 14.6 | 15.8 | 16.8 | 17.8 |
| 16 | 9.5 | 10.1 | 10.8 | 11.7 | 12.4 | 12.9 | 13.4 | 14.0 | 14.6 | 15.4 | 16.6 | 17.6 | 18.5 |
| 17 | 10.2 | 10.8 | 11.5 | 12.4 | 13.1 | 13.6 | 14.2 | 14.8 | 15.4 | 16.2 | 17.2 | 18.1 | 18.9 |
| 18 | 11.0 | 11.5 | 12.2 | 13.1 | 13.8 | 14.4 | 15.0 | 15.5 | 16.2 | 16.9 | 17.8 | 18.6 | 19.2 |
| 19 | 11.7 | 12.3 | 12.9 | 13.8 | 14.5 | 15.1 | 15.7 | 16.3 | 16.8 | 17.5 | 18.3 | 18.9 | 19.4 |
| *Females* |  |  |  |  |  |  |  |  |  |  |  |  |  |
| 6 | 5.1 | 5.4 | 5.9 | 6.3 | 6.7 | 7.0 | 7.3 | 7.6 | 8.1 | 8.7 | 9.6 | 10.4 | 11.2 |
| 7 | 5.4 | 5.7 | 6.1 | 6.6 | 6.9 | 7.3 | 7.6 | 7.9 | 8.3 | 8.9 | 9.7 | 10.4 | 11.1 |
| 8 | 5.6 | 6.0 | 6.4 | 6.9 | 7.3 | 7.6 | 7.9 | 8.3 | 8.7 | 9.3 | 10.1 | 10.8 | 11.4 |
| 9 | 5.9 | 6.3 | 6.7 | 7.3 | 7.7 | 8.0 | 8.4 | 8.8 | 9.2 | 9.7 | 10.5 | 11.2 | 11.9 |
| 10 | 6.2 | 6.6 | 7.1 | 7.7 | 8.1 | 8.4 | 8.8 | 9.2 | 9.7 | 10.3 | 11.1 | 11.8 | 12.4 |
| 11 | 6.5 | 6.9 | 7.4 | 8.0 | 8.5 | 8.9 | 9.3 | 9.7 | 10.2 | 10.8 | 11.7 | 12.4 | 13.1 |
| 12 | 6.8 | 7.3 | 7.8 | 8.4 | 8.9 | 9.3 | 9.8 | 10.2 | 10.7 | 11.4 | 12.2 | 13.0 | 13.7 |
| 13 | 7.1 | 7.6 | 8.1 | 8.8 | 9.3 | 9.8 | 10.2 | 10.7 | 11.2 | 11.9 | 12.8 | 13.6 | 14.3 |
| 14 | 7.3 | 7.8 | 8.5 | 9.2 | 9.7 | 10.2 | 10.6 | 11.1 | 11.6 | 12.3 | 13.2 | 14.0 | 14.8 |
| 15 | 7.5 | 8.1 | 8.7 | 9.5 | 10.0 | 10.5 | 11.0 | 11.5 | 12.0 | 12.7 | 13.6 | 14.4 | 15.2 |
| 16 | 7.7 | 8.3 | 9.0 | 9.8 | 10.3 | 10.8 | 11.2 | 11.7 | 12.3 | 12.9 | 13.9 | 14.7 | 15.4 |
| 17 | 7.8 | 8.4 | 9.1 | 9.9 | 10.5 | 11.0 | 11.4 | 11.9 | 12.4 | 13.0 | 14.0 | 14.8 | 15.6 |
| 18 | 7.8 | 8.5 | 9.2 | 10.0 | 10.6 | 11.1 | 11.5 | 11.9 | 12.4 | 13.0 | 14.0 | 14.8 | 15.5 |
| 19 | 7.7 | 8.4 | 9.1 | 10.0 | 10.6 | 11.0 | 11.4 | 11.8 | 12.3 | 12.9 | 13.8 | 14.6 | 15.3 |

*Notes:* Norm-referenced values are in kg/m^2^; population-weighted smoothed percentiles were calculated using the Generalized Additive Model for Location, Scale and Shape method; the ages shown represent age (e.g., 6 = 6.00–6.99…) based on age at last birthday.

Abbreviations: *P* percentile (e.g., P5 = 5^th^ percentile), *y* years.

**ELECTRONIC SUPPLEMENTARY MATERIAL**

**Article title:** How *should* youth handgrip strength be normalized? New insights using 3-D allometry with “generalizable” norm-referenced values, data from NHANES; **Journal name:** Sports Medicine; **Author names and affiliations:** Alan M Nevill (University of Wolverhampton), Justin J. Lang (Public Health Agency of Canada, University of Ottawa, and University of South Australia), Mark Niemz (University of Wolverhampton), Grant R. Tomkinson (University of South Australia); **E-mail address of the corresponding author:** [grant.tomkinson@unisa.edu.au](mailto:grant.tomkinson@unisa.edu.au).

**Figure S1.** Percentile curves for normalized handgrip strength (handgrip strength in kilograms [kg] divided by height in meters [m] squared) by age for (a) males and (b) females.


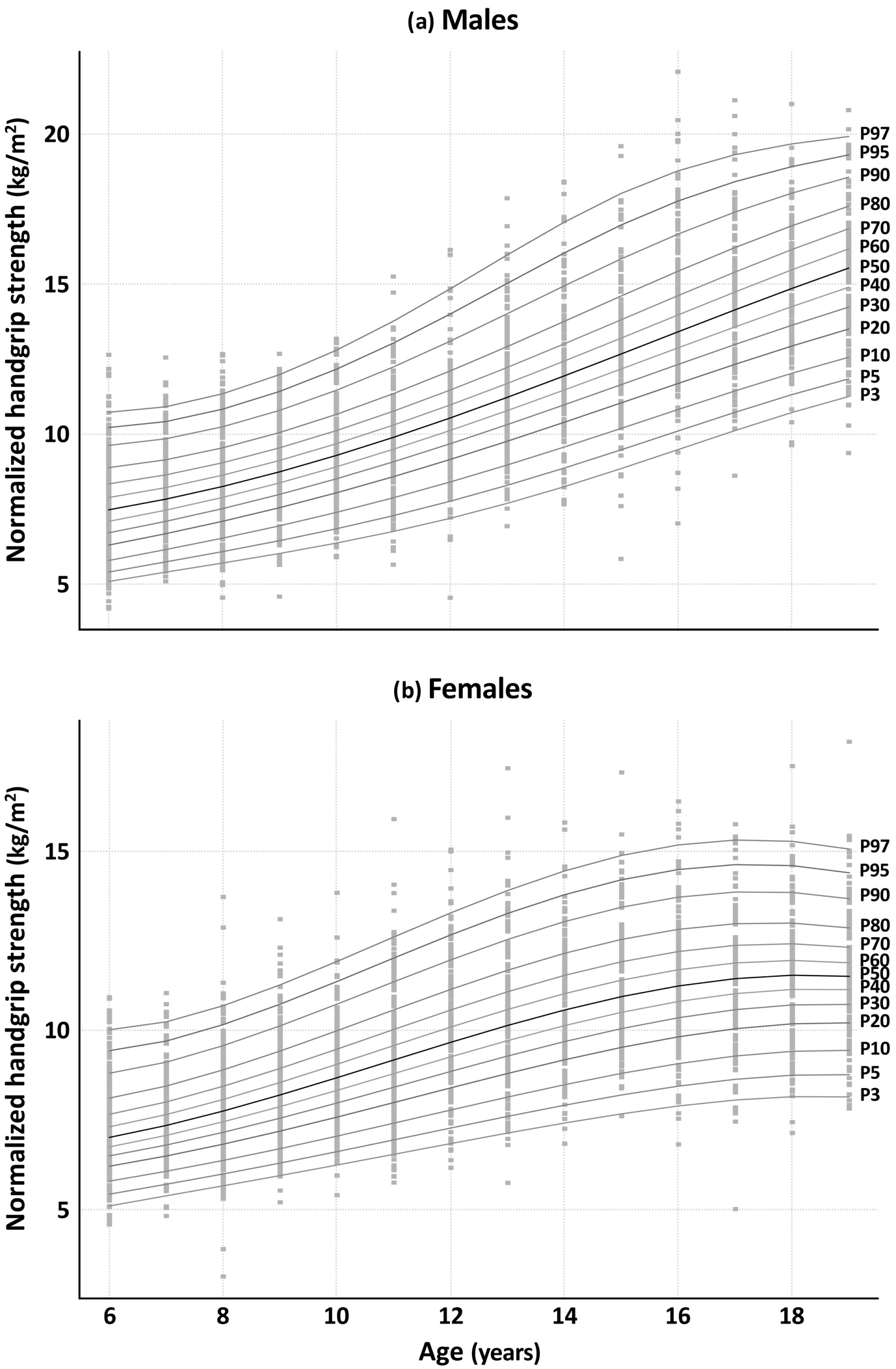

Supplement: Supplementary file 1 — Supplementary file1 (DOCX 470 KB) [file 40279_2025_2235_MOESM1_ESM.docx]
